# Phacoemulsification in bilateral anterior lenticonus in Alport syndrome: A case report

**DOI:** 10.1097/MD.0000000000017054

**Published:** 2019-09-27

**Authors:** Mohammad-Reza Sedaghat, Hamed Momeni-Moghaddam, Batool Haghighi, Majid Moshirfar

**Affiliations:** a Eye Research Center, Mashhad University of Medical Sciences, Mashhad; b Health Promotion Research Center, Zahedan University of Medical Sciences, Zahedan; c Department of Optometry, School of Paramedical Sciences, Mashhad University of Medical Sciences, Mashhad, Iran; d John A. Moran Eye Center, Department of Ophthalmology and Visual Sciences, School of Medicine, University of Utah; e Utah Lions Eye Bank, Murray; f HDR Research Center, Hoopes Vision, Draper, UT.

**Keywords:** Alport syndrome, anterior segment optical coherence tomography, cataract, crystalline lens, lenticonus

## Abstract

**Rationale:**

To report the visual status and results of phacoemulsification cataract surgery in a young patient with Alport syndrome associated with bilateral anterior lenticonus. The milestone of this report is the use of anterior segment optical coherence tomography (AS-OCT) to confirm the central protrusion of the anterior surface of the crystalline lens.

**Patient concerns:**

A 23-year-old young woman presented with severe progressive visual loss in both eyes, which started several years ago.

**Diagnoses:**

Refractive status was indicative of high myopia with astigmatism and vision was not improved with optimal correction to better than 0.1 in the right eye and 0.2 in the left eye (visual acuities given in decimal notation). Slit-lamp examination showed transparent cornea, anterior lenticonus and posterior sub-capsular cataract in both eyes. The classical appearance of oil droplet was evident using retro-illumination on the slit lamp.

**Interventions:**

The natural lenses were replaced with intraocular lens (IOL).

**Outcomes:**

An excellent refractive status achieved associated with an uncorrected distance visual acuity 0.9 and 0.8 in the right and left eye, respectively.

**Lessons:**

AS-OCT is a valuable device for confirming the budging of the anterior crystalline lens surface.

## 1 Introduction

Lenticonus is an uncommon condition where the crystalline lens cortex and the overlying capsule acquire a localized, cone-shaped deformation in one or both lens surfaces.^[1]^ Bilateral anterior lenticonus is commonly seen in Alport syndrome (AS), which is a hereditary (X- linked inheritance as the most common pattern) and progressive disease caused by mutation in the gene of the basement membrane collagen type IV. It is characterized by nephritis, and ocular and auditory anomalies.^[2]^ Major ocular manifestations are anterior lenticonus (usually axial), fleck retinopathy, corneal opacity, and cataract.^[3,4]^ Progressive visual deterioration over the time is mainly due to induced myopia and astigmatism secondary to lenticonus. However, visual quality may also be affected by the bulging of the anterior lens capsule that generates increasing levels of aberrations. In a symptomatic patient with no cataract or very mild cataract, clear lens extraction (CLE) is a good option for replacing the inefficient lens^[5]^ and this is also the case in the presence of a cataract, where an intra-ocular lens (IOL) will improve visual quantity and quality by replacing the distorted refractive component.

## 2 Case presentation

**Figure 1:**
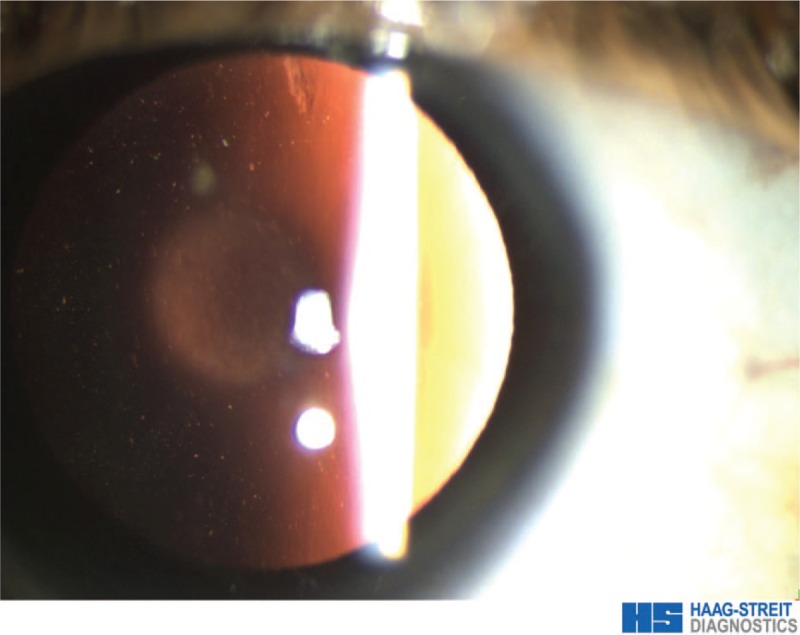
Oil droplet appearance on retro-illumination from fundus.

A 23-year-old woman presented with progressive reduced vision in both eyes, starting several years ago. Informed written consent was obtained from the patient for publication of this case report and accompanying images. Uncorrected Distance visual acuity (UDVA) and best corrected distance visual acuity (CDVA) were was counting fingers (CF) and 0.1 (decimal notation), respectively, with a refraction –8.00 / –5.25 × 24° in the right eye (RE), and CF, and 0.2, respectively, with a refraction –10.50 / –2.75 × 168° in the left eye (LE). Slit lamp examinations revealed normal cornea, anterior lenticonus, oil droplet appearance (Fig. 1) and posterior sub-capsular cataract in both eyes.

**Figure 2:**
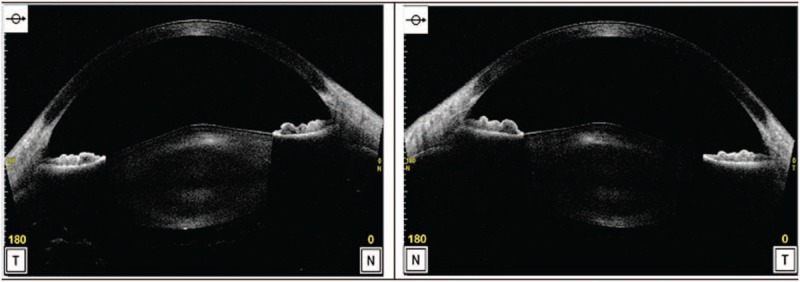
OCT images of anterior lenticonus in the right eye (left panel) and the left eye (right panel).

Association of anterior lenticonus with renal and hearing abnormalities was indicative of AS. The use of anterior segment optical coherence tomography CASIA2 (Tomey Corporation, Nagoya, Japan) to display better and confirm the diagnosis of anterior lenticonus has not been used in previous published papers (Fig. 2).

Intra-ocular pressure was 12 mmHg in both eyes using Topcon non-contact tonometer (Topcon Corporation, Tokyo, Japan). Dilated fundus examination showed normal retina and optic disc with a cup-to-disc ratio of 50% in both eyes. The corneal topography using TMS-4 (TOMEY Corporation, Nagoya, Japan) showed normal pattern and indices, with simulated keratometry of 45.5/44.18@79 and 45.11/43.79@160 in the RE and the LE, respectively. Axial length, anterior chamber depth and crystalline lens thickness were 23.74, 3.47, 3.20 mm in the RE and 23.86, 3.43, 3.48 mm in the LE, respectively, all of them measured using IOLMaster 700 (Carl Zeiss Meditec AG, Jena, Germany). Comparison of total (refractive) and corneal astigmatism in the right (5.25DC, 1.31DC, respectively) and the LE (2.75DC, 1.32DC, respectively) showed that the essential component of astigmatism was lenticular (3.94D in the RE and 1.43D in the LE). The patient's chief complaint was blurred vision. The abnormal crystalline lenses were extracted sequentially first in the RE and then one month later in the LE using phacoemulsification method. The distorted crystalline lenses were replaced with hydrophobic acrylic mono-focal IOLs (enVista MX60, Bausch & Lomb, Rochester, NY) with powers of +19.00D. Post-operative therapeutic regimen was an antibiotic eye drop (oftaquix), a topical corticosteroid (betamethasone 0.1%) with a decreasing dosage. Four weeks after surgery, UDVA was 0.9 in the RE with a refraction +0.50 / –1.00 × 180° and 0.8 with a refraction 0.00 / –1.00 × 163° in the left eye. Post-refractive total and corneal astigmatisms (right KR: 45.25/44@180 & left KR: 43.5/44.75@164) were similar and this indicated that the lenticular astigmatic component had been totally removed. Post-surgical uncorrected near vision was 0.2 in both eyes, increasing to 0.9 with a reading addition of +2.00 for a near distance of 40 cm.

## 3 Discussion

AS is an uncommon disorder characterized with ocular anomalies, progressive nephritis and sensorineural hearing difficulties. Development and progress of myopia and astigmatism are secondary to structural changes in one or both lenticular surfaces.^[3,4]^

Various anterior segment analyzers, such as ultrasound biomicrospe (UBM), Scheimpflug-based tomographers and systems compounding Scheimpflug and Placido-disk based technologies are available on the market; however, the importance of the AS-OCTs in assessing the anterior segments parameters is not overlooked.^[6]^ Compared to the AS-OCT, UBM is a manually guided contact measurement that has low reproducibility, very poor axial and lateral resolution, no 3-dimentional imaging option, no automatic image processing and image analysis systems. Apart from the advantages of the rotating Scheimpflug systems, this technology uses a blue light source with a wavelength of about 475 nm which has very limited penetration in opaque optical media, in addition; large refractive index difference from air to cornea provides a strong scatter and reflection at corneal front surface, which challenges edge detection. While the spectral and swept-source OCT systems have high scanning rate, high lateral and axial resolutions, simultaneous acquisition of depth information and fast 3-dimentional scanning.^[7]^

AS-OCT has been used in exploring the anterior segment parameters, mainly the cornea and anterior chamber characteristics (depth, angle, and volume), but some of them are able to evaluate other structures of the anterior segment, such as iris characteristics and features of the crystalline lens.^[8,9]^ Comparing the depth measurement range from front corneal surface to posterior lens surface and anterior portion of the vitreous body of different AS-OCTs including Visante (Carl Zeiss Meditec, Inc., Dublin, CA), RTVue (Optovue, Inc., Fremont, California), Cirrus 5000 HD (Carl Zeiss Meditec, Inc., Dublin, CA), Casia SS-1000 (Tomey Corporation, Nagoya, Japan), Casia2 (Tomey Corporation, Nagoya, Japan) indicates that the CASIA2 as a Swept-source OCT provides the highest depth rage (approximately 13 mm) which allows for visualization of the entire lens and a better overview analysis of lens shape, position and orientation.^[10,11]^ Therefore, this feature enables the device to evaluate the localized curvature changes in the shape of both lens surfaces, such as lenticonus.

In the present case, lenticular structural changes were associated with different amounts of induced lenticular astigmatism, 3.94D in the RE and 1.43D in the LE in our case. Although satisfactory outcomes have been reported following CLE in these patients,^[12,13]^ in our case there was posterior sub-capsular cataract with significant visual impairment, hence phacoemulsification cataract surgery with IOL implantation was selected as the therapeutic option. Monofocal IOL was selected as an appropriate option with attention to the lenticular component as the main part of pre-operative astigmatism. There is possibility of correction of corneal astigmatism, especially lower amounts of against-the-rule and oblique astigmatism, with other methods, such as LRI (limbal relaxing incisions) and AK (astigmatic keratotomy).^[14,15]^

Failure to improve the pre-operative UDVA to better than 0.1 and 0.2 with optimal correction may direct the practitioner to suspect refractive amblyopia.^[5]^ However, the presence or absence of lenticonus should be also investigated. In our case, the poor vision was not only due to lenticonus as this patient had the additional presence of significant cataract.

One notable point in these patients is that decreased thickness of anterior capsule secondary to the conical shape of crystalline lens makes the anterior capsule extremely flexible and fragile. Therefore, capsulorhexis is very difficult technically in these patients so the use of microforceps is recommended to prevent the peripheral extension of capsulorhexis.^[16]^

## 4 Conclusion

AS involves multiple organs, including kidney, ear, and eye. One of ocular manifestation is anterior lenticonus in which a budging or localized curvature change occurring in the anterior crystalline lens surface can be best viewed in the AS-OCT and it may use to make the patient aware of the change in his eye structure. These changes may cause slow progressive deterioration of vision requiring patients to change their prescription frequently due to changes in the magnitude of induced myopic astigmatism as well as cataracts. To restore visual capacity, CLE or cataract surgery may be indicated based on the status of crystalline lens transparency.

## Acknowledgments

The authors would like to thank the subject and personals of Sedaghat eye clinic.

## Author contributions

**Conceptualization:** Mohammad-Reza Sedaghat, Hamed Momeni-Moghaddam, Batool Haghighi, Majid Moshirfar.

**Methodology:** Mohammad-Reza Sedaghat.

**Project administration:** Hamed Momeni-Moghaddam, Batool Haghighi, Majid Moshirfar.

**Supervision:** Mohammad-Reza Sedaghat, Hamed Momeni-Moghaddam.

**Writing – original draft:** Mohammad-Reza Sedaghat, Hamed Momeni-Moghaddam, Batool Haghighi, Majid Moshirfar.

**Writing – review & editing:** Mohammad-Reza Sedaghat, Hamed Momeni-Moghaddam, Batool Haghighi, Majid Moshirfar.
